# Isodeoxyelephantopin Promotes Cell Death in Triple‐Negative Breast Cancer by Blocking STAT3 Phosphorylation and Enhancing the Effectiveness of Paclitaxel

**DOI:** 10.1002/fsn3.70737

**Published:** 2025-09-05

**Authors:** Danfeng Lin, Hui Wang, Shixi Wu, Haha Chen

**Affiliations:** ^1^ Department of Breast Surgery The First Affiliated Hospital of Wenzhou Medical University Wenzhou Zhejiang China; ^2^ Department of Pathology, Zhejiang Cancer Hospital Hangzhou Institute of Medicine, Chinese Academy of Sciences Hangzhou Zhejiang China; ^3^ Department of Thyroid and Breast Surgery, Oncological Surgery The Third Affiliated Hospital of Wenzhou Medical University Wenzhou Zhejiang China

**Keywords:** combination treatment, isodeoxyelephantopin, paclitaxel, signal transducer and activator of transcription 3, triple‐negative breast cancer

## Abstract

Triple‐negative breast cancer (TNBC) is an aggressive and challenging subtype of breast cancer, presenting patients with a more complex treatment journey. This underscores the critical need for ongoing research and the development of effective therapies to enhance patient outcomes. Signal transducer and activator of transcription 3 (STAT3) is a crucial transcription factor that regulates various cellular processes, including proliferation, survival, and immune modulation. Its constitutive activation is frequently observed in multiple cancer types, contributing to tumor progression and immune evasion. Isodeoxyelephantopin (IDET) is a bioactive compound extracted from the traditional medicinal plant *Elephantopus scaber* , yet its anti‐tumor mechanisms require further investigation for a comprehensive understanding. The expression of indicated proteins was detected by Western blot analysis. Viability was evaluated using the trypan blue exclusion test. The combination index (CI) values were determined using CompuSyn software, based on the Chou‐Talalay method. The anti‐tumor activity of IDET combined with paclitaxel in vivo was confirmed in nude mice. In this study, we initially discovered that inhibiting STAT3 phosphorylation plays a vital role in the anti‐tumor activity of IDET against TNBC. Furthermore, we found that IDET can enhance the anti‐tumor activity of cisplatin and paclitaxel. Mechanistically, the inhibition of STAT3 phosphorylation is pivotal in mediating the synergistic anti‐tumor effects observed with the combination of IDET and paclitaxel. Importantly, IDET also enhances the anti‐tumor activity of paclitaxel in vivo. Taken together, our study reveals a novel mechanism of IDET and provides a potential strategy for treating TNBC.

## Graphical abstract

This study demonstrates that isodeoxyelephantopin (IDET) inhibits triple‐negative breast cancer (TNBC) by suppressing STAT3 phosphorylation. This inhibition is also crucial for the enhanced efficacy observed when IDET is combined with paclitaxel. IDET also enhances the anti‐tumor efficacy of paclitaxel in vivo, with the combined treatment showing significant tumor suppression and good tolerance. These findings highlight the potential of IDET as a therapeutic agent and provide a rationale for its combination with paclitaxel in TNBC treatment.

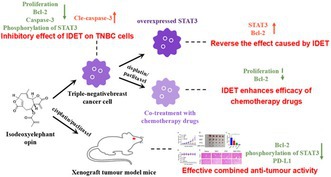

## 1 Introduction

Triple‐negative breast cancer (TNBC) is a highly aggressive subtype of breast cancer characterized by the absence of three essential receptors: estrogen, progesterone, and human epidermal growth factor receptor 2 (HER2) (Bianchini et al. 2022; Wang, Hao, et al. 2024). This lack of receptors results in TNBC's resistance to standard treatments effective for other breast cancer types, including hormone therapies and HER2‐targeted therapies. Consequently, patients with TNBC often face a more difficult treatment journey, with limited options primarily revolving around chemotherapy and emerging targeted therapies (Harris et al. 2024; Zhu et al. 2023). The aggressive nature of TNBC, coupled with a higher risk of recurrence and poorer overall prognosis compared to other breast cancer subtypes, underscores the urgent need for ongoing research and development of effective treatments to improve outcomes (Choupani et al. 2023; Lam et al. 2023).

STAT3 is a vital transcription factor that plays a key role in cellular signaling and the regulation of gene expression. In cancer research, it has become a central focus due to its involvement in tumorigenesis, cancer progression, and drug resistance (Wang et al. 2022; Kim et al. 2024). STAT3 is often constitutively activated in various cancers, including breast, lung, and hematological malignancies, with this aberrant activation typically arising from mutations, overexpression of upstream cytokine receptors, or dysregulation of related signaling pathways (He et al. 2022; Wong et al. 2022; Brandstoetter et al. 2023). Activated STAT3 facilitates oncogenesis by promoting the expression of genes that enhance cell proliferation, survival, and angiogenesis, while simultaneously inhibiting apoptosis and immune surveillance (Wu et al. 2023; Li et al. 2022). Its role in creating an immunosuppressive tumor microenvironment further complicates treatment, reducing the effectiveness of immune‐based therapies (Alcantara et al. 2023; Zou et al. 2020). Consequently, targeting STAT3 has emerged as a prominent strategy in cancer therapy, with researchers developing small molecules, peptides, and monoclonal antibodies designed to disrupt its activity or inhibit its interactions (Wang, Liao, et al. 2024; Mohan et al. 2022; Fan et al. 2024). Understanding the precise role of STAT3 in different cancer types remains essential for advancing therapeutic approaches and enhancing patient outcomes.

Isodeoxyelephantopin is a bioactive sesquiterpene lactone isolated from the medicinal plant *Elephantopus scaber* , which is well‐known for its traditional therapeutic applications (Ichikawa et al. 2006). Recently, this compound has garnered considerable interest due to its diverse pharmacological properties (Han et al. 2020; Mehmood and Muanprasat 2022). Research has highlighted its potential benefits in treating inflammatory conditions and cancer, with studies demonstrating its ability to induce apoptosis in tumor cells, inhibit cell proliferation, and regulate oxidative stress, activate the JNK signaling pathway, suppress NF‐κB activation, and induce cell cycle arrest (Hong et al. 2020; Verma et al. 2019; Kabeer et al. 2014). However, its anti‐tumor mechanism still needs further investigation.

In our study, we initially found that inhibiting the phosphorylation of STAT3 is essential for the anti‐tumor effects of IDET against TNBC. This inhibition appears to be a key factor in IDET's ability to combat TNBC effectively. Additionally, our research demonstrated that IDET possesses inherent anti‐tumor activity and significantly enhances the effectiveness of conventional chemotherapy agents, including cisplatin and paclitaxel. From a mechanistic perspective, the inhibition of STAT3 phosphorylation is crucial for the synergistic anti‐tumor effects observed when combining IDET with paclitaxel. Importantly, our in vivo experiments confirmed that IDET enhances paclitaxel's anti‐tumor activity, reinforcing the potential of this combination approach. Overall, our study uncovers a novel mechanism through which IDET operates and highlights its potential as part of a strategic approach to treating TNBC.

## 2 Materials and Methods

### 2.1 Chemicals and Reagents

Isodeoxyelephantopin was acquired from DESITE Company (#DY0935‐0020, purity ≥ 98%; Chengdu, China). Isodeoxyelephantopin was dissolved in DMSO, aliquoted into small tubes, and stored at −80°C. The final concentration of DMSO in the solutions was maintained at or below 0.1% during use. Antibodies including p‐STAT3 (#9145) and STAT3 (#30835) were purchased from Cell Signaling Technology (Danvers, USA). Antibodies including Bcl‐2 (#12789–1‐AP), Caspase‐3 (#19677–1‐AP), GAPDH (#60004–1‐Ig), and secondary antibodies (#SA00001‐2, #SA00001‐1) were purchased from Proteintech (Wuhan, China).

### 2.2 Cell Culture

MDA‐MB‐231 (#TCHu227), BT‐549 (#TCHu93), and MCF‐10A (#SCSP‐575) cell lines were purchased from the Cell Bank of Chinese Academy of Sciences (Shanghai, China). The cells underwent STR profiling and mycoplasma testing, both of which were confirmed to be accurate. MDA‐MB‐231 cells were cultured in Dulbecco's Modified Eagle's Medium (DMEM, #C11995500BT; Gibco, USA) containing 10% fetal bovine serum (FBS, #10099141C; Gibco, USA) at 37°C. BT‐549 cells were cultured in RPMI 1640 medium (#C11875500BT; Gibco, USA) containing 10% FBS at 37°C. MCF‐10A cells were maintained in DMEM/F12 medium (#C11330500BT; Gibco, USA) supplemented with a MEGM Kit (#CC‐3150; Lonza, Switzerland) at 37°C. Cell passage was conducted on a meticulously maintained clean platform to ensure sterility. After the digestion process and subsequent centrifugation, the cells were carefully resuspended in complete medium. They were then plated onto appropriate cell culture dishes or plates for optimal growth. The cultures were incubated in a humidified atmosphere at 37°C to promote cell proliferation and viability.

### 2.3 Cell Viability

Cells were carefully seeded into six‐well plates (#3516; Corning, USA) at a density of 2 × 10^5^ cells per well in 2 mL of complete medium and left to incubate overnight to promote proper attachment to the culture surface. Following this incubation period, the medium was replaced with fresh complete medium to ensure optimal growth conditions. The cells were then treated with a range of concentrations of isodeoxyelephantopin, administered either as a single agent or in combination with cisplatin (#P4394; Sigma‐Aldrich, USA) and paclitaxel (#T7402; Sigma‐Aldrich, USA), for 24 h. Viability was evaluated using the trypan blue exclusion test (#T8154; Sigma‐Aldrich, USA). The Combination Index (CI) values were determined using CompuSyn software (ComboSyn Inc., USA), with a CI value below 1 indicating a synergistic interaction (Chou 2010).

### 2.4 Western Blot Analysis

Cells were lysed using a lysis buffer (RIPA Buffer, #89900; Thermo Fisher Scientific, USA) specifically formulated to extract total proteins effectively. After lysis, the protein samples were carefully combined with a 5× loading buffer (#LC2676; Thermo Fisher Scientific, USA), and then heated in a boiling water bath for a duration of 10 min to facilitate proper denaturation of the proteins, ensuring that they will migrate accurately during the separation process. Following the heating step, the samples were subjected to separation through SDS‐PAGE. Once the separation was complete, the proteins were transferred onto a PVDF membrane (#IPVH00010; Merck Millipore, USA) and incubated overnight with a specific primary antibody. After completing three washes with TBST, each lasting 5 min to ensure thorough removal of any unbound components, the membrane was then treated with a secondary antibody for a duration of 1 h. Following the incubation with the secondary antibody, signal detection was performed using a high‐sensitivity ECL substrate kit (#34095; Thermo Fisher Scientific, USA). Finally, the intensity of the resulting signals was meticulously measured and quantified using ImageJ software.

### 2.5 RNA Extraction and qRT‐PCR

Total RNA was isolated from tissues and cells using TRIzol reagent (#T9424; Sigma‐Aldrich, USA) according to the manufacturer's protocol. Using reverse transcription kit (#RR047A; Takara, Japan), we reverse transcribed RNA into cDNA. All primer sequences are listed in Table 1. The internal control (GAPDH) was used to normalize mRNA expression levels.

**TABLE 1:** Primer sequences.

Primer	Sequence (5′‐3′)
STAT3	F: CTTTGAGACCGAGGTGTATCACC R: GGTCAGCATGTTGTACCACAGG
PD‐L1	F: ACCCAGAAGACTGTGGATGG R: TGCAGCCAGGTCTAATTGTTTT
Bcl‐2	F: ATCGCCCTGTGGATGACTGAGT R: GCCAGGAGAAATCAAACAGAGGC
GAPDH	F: GGCAACCCTGTGCTACGAAT R: TCAGCTCAGGGATGACCTTG

### 2.6 Animal Model

In this study, female BALB/c nude mice (6–8 weeks, healthy, SPF‐grade) were carefully housed in the designated experimental animal facility, ensuring optimal conditions for their well‐being and health. The use of these mice was granted approval by the ethical committee of the First Affiliated Hospital of Wenzhou Medical University (WYYY‐IACUC‐AEC‐2024‐120). The mice were injected subcutaneously with tumor cells that had been mixed with matrigel (#354234; Corning, USA). Once the tumors reached a suitable size, the mice were divided into four treatment groups: a vehicle group, a group receiving isodeoxyelephantopin at a dosage of 5 mg/kg, a group receiving paclitaxel at a dosage of 1 mg/kg, and a group receiving a combination of both isodeoxyelephantopin at 5 mg/kg and paclitaxel at 1 mg/kg. Tumor sizes and body weights were recorded every other day throughout the study. At the end of the experimental period, the mice were euthanized, and cancer tissues, along with vital organs such as the liver and kidneys, were collected for further analysis, including immunoblotting assays and hematoxylin and eosin staining (#C0105S; Beyotime, China).

### 2.7 Statistical Analysis

Statistical analysis of the data was performed using GraphPad Prism 6.0 software. To assess differences between the experimental groups, either the Student's *t*‐test or one‐way ANOVA was utilized. A *p*‐value of less than 0.05 was considered indicative of a statistically significant difference.

## 3 Results

### 3.1 IDET Can Inhibit the Growth of TNBC Cells in a Dose‐Dependent Manner

**FIGURE 1:**
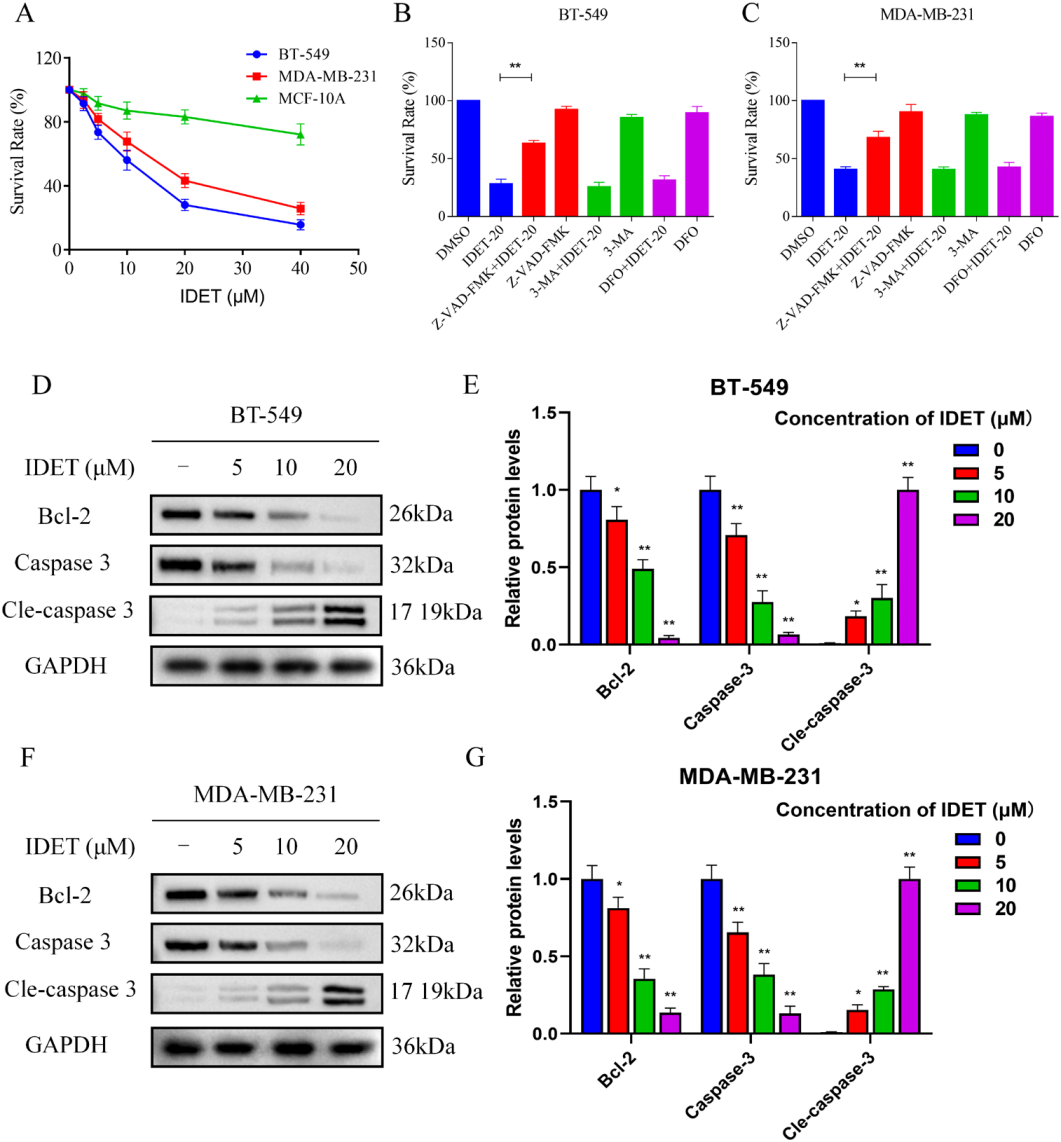
Isodeoxyelephantopin (IDET) suppresses the growth of triple‐negative breast cancer (TNBC) cells in a dose‐dependent manner. (A) The survival rate of cells after treatment with different concentrations of IDET for 24 h was assessed. (B, C) The cells were pretreated with Z‐VAD‐FMK, 3‐MA, or DFO for 1 h, and then the survival rate was assessed after treatment with IDET for 24 h. (D, F) The levels of Bcl‐2, Caspase 3, and Cle‐caspase 3 in BT‐549 and MDA‐MB‐231 cells were measured following treatment with IDET. (E, G) Statistical analysis of western blot results for D and F.

We initially evaluated IDET's inhibitory effects on TNBC cells. Our findings revealed that IDET effectively reduced the proliferation of MDA‐MB‐231 and BT‐549 cells in a concentration‐dependent manner. Importantly, IDET showed only minimal impact on the proliferation of normal MCF‐10A cells, demonstrating its selective action (Figure 1A). To explore the potential cell death mechanisms of IDET in TNBC cells, we used Z‐VAD‐FMK (an apoptosis inhibitor), 3‐Methyladenine (an autophagy inhibitor), and deferoxamine mesylate (a ferroptosis inhibitor). The results showed that Z‐VAD‐FMK significantly reversed the anti‐tumor activity of IDET (Figure 1B,C). Western blotting analysis revealed that IDET significantly decreased the expression of apoptosis‐related proteins Bcl‐2 and Caspase‐3, while increasing the levels of cleaved Caspase‐3 in TNBC cells, indicating that IDET exerts its anti‐tumor effect primarily through the induction of apoptosis in TNBC cells (Figure 1D–G).

### 3.2 The Inhibition of STAT3 Phosphorylation Is Vital for the Anti‐Tumor Activity of IDET

**FIGURE 2:**
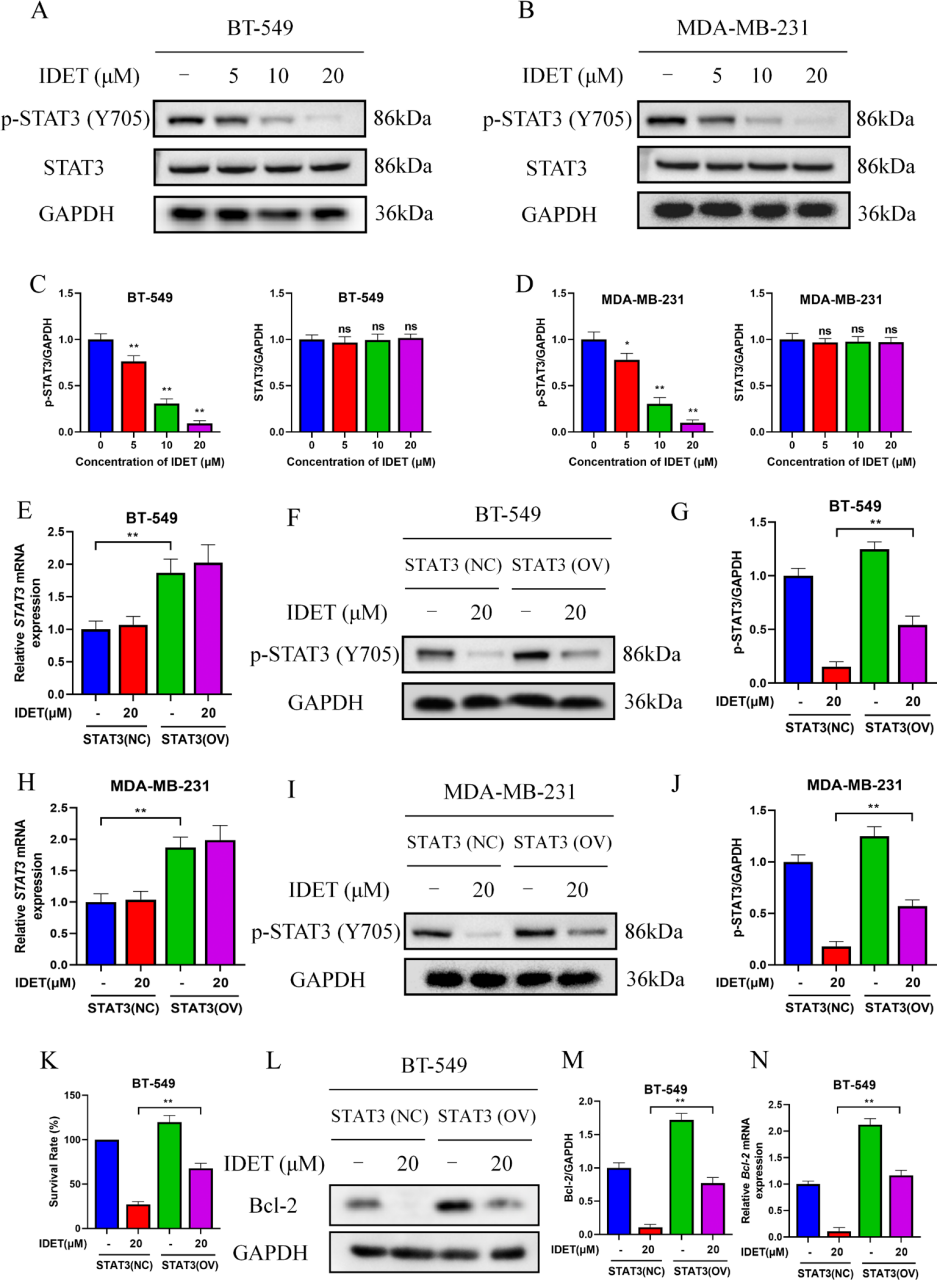
The inhibition of signal transducer and activator of transcription 3 (STAT3) phosphorylation is vital for the anti‐tumor activity of Isodeoxyelephantopin (IDET). (A, B) The levels of p‐STAT3 and STAT3 in BT‐549 and MDA‐MB‐231 cells were assessed following treatment with IDET. (C, D) Statistical analysis of western blot results for A and B. (E, H) The mRNA expression levels of STAT3 in BT‐549 and MDA‐MB‐231 cells, both overexpressing STAT3, were measured following IDET treatment. (F, G, I, J) The levels of p‐STAT3 in BT‐549 and MDA‐MB‐231 cells, both overexpressing STAT3, were measured following treatment with IDET. (K) The survival rate of cells was assessed after treatment with IDET for 24 h. (L–N) The levels of Bcl‐2 in BT‐549 (overexpressing STAT3) cells were measured following treatment with IDET.

STAT3 is frequently found to be constitutively active in various cancer cells, including TNBC cells (Long et al. 2024; Mahata et al. 2022). Interestingly, we discovered that IDET can inhibit the phosphorylation of STAT3 in a concentration‐dependent manner (Figure 2A–D). To further confirm that the STAT3 pathway mediates the anti‐tumor activity of IDET, we overexpressed STAT3 in TNBC cells. Western blotting results revealed that the phosphorylation level of STAT3 significantly increases when STAT3 is overexpressed in MDA‐MB‐231 and BT‐549 cells (Figure 2E–J). Importantly, cells with high expression of STAT3 exhibited a significant decrease in sensitivity to IDET, and the reduction in Bcl‐2 caused by IDET was also reversed (Figure 2K–N).

### 3.3 IDET Can Enhance the Anti‐Tumor Activity of Cisplatin in TNBC Cells

**FIGURE 3:**
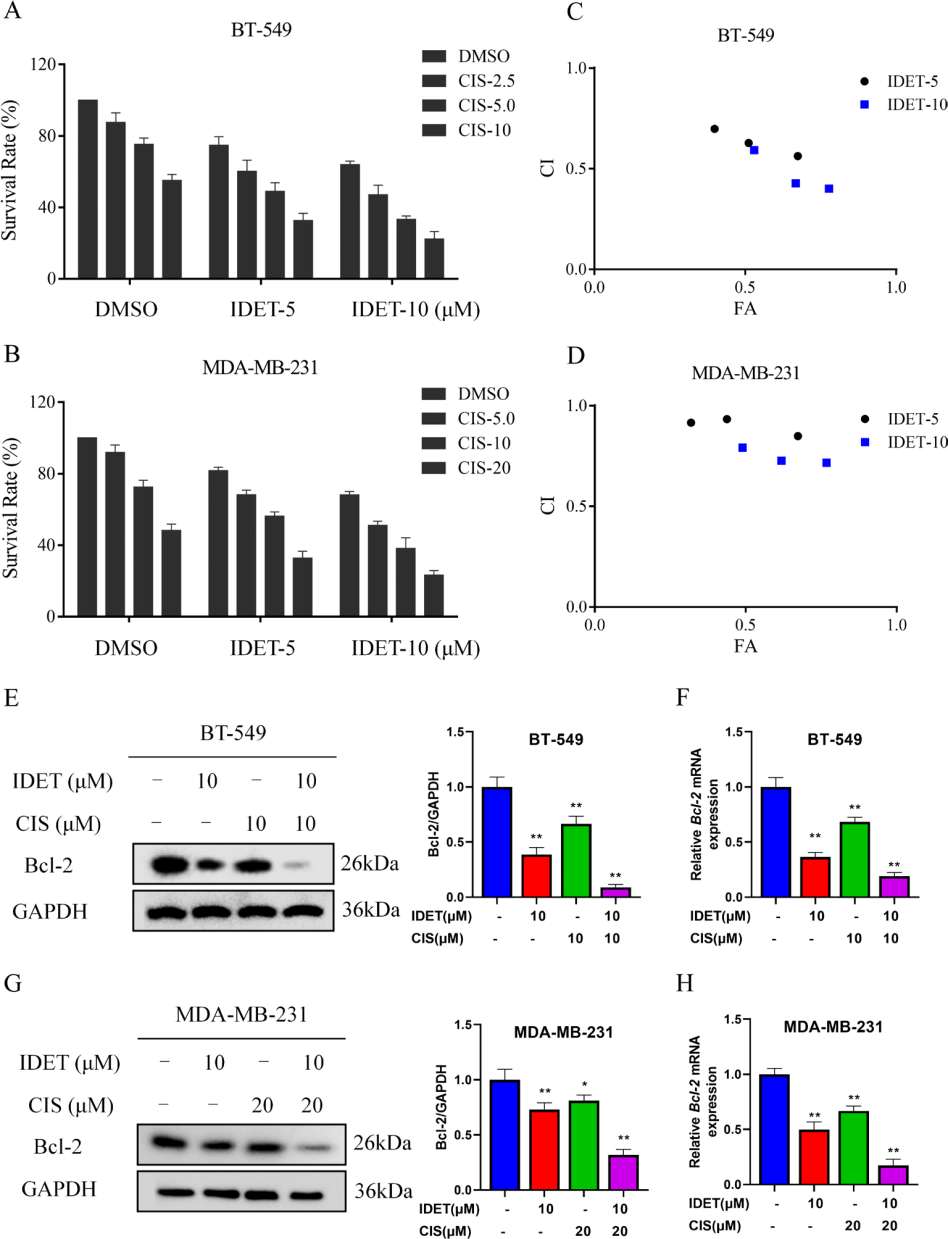
Isodeoxyelephantopin (IDET) enhances the anti‐tumor activity of cisplatin in triple‐negative breast cancer (TNBC) cells. (A, B) The survival rate of cells treated for 24 h with various concentrations of CIS, either alone or in combination with IDET, was evaluated. (C, D) The corresponding CI values were calculated based on the survival rate results shown in panels A and B. (E–H) The levels of Bcl‐2 in BT‐549 and MDA‐MB‐231 cells were evaluated after treatment with IDET, CIS, or their combination.

Previous research indicates that blocking the STAT3 signaling pathway can potentiate the efficacy of anti‐cancer therapies (Zuo et al. 2024; Zhong et al. 2024; Wang, Shi, et al. 2023). Cisplatin and paclitaxel are both significant chemotherapeutic agents. Therefore, we first examined the anti‐TNBC effects of IDET in combination with cisplatin. Survival experiments revealed that combining IDET with cisplatin inhibits cell growth significantly more effectively than using either IDET or cisplatin alone (Figure 3A,B). The combination index values further indicate that IDET and cisplatin display synergistic anti‐tumor activity in TNBC cells (Figure 3C,D). Western blotting and qRT‐PCR results revealed that the combination of IDET and cisplatin more effectively inhibited the expression of Bcl‐2 compared to monotherapy (Figure 3E–H).

### 3.4 IDET Can Enhance the Anti‐Tumor Activity of Paclitaxel in TNBC Cells

**FIGURE 4:**
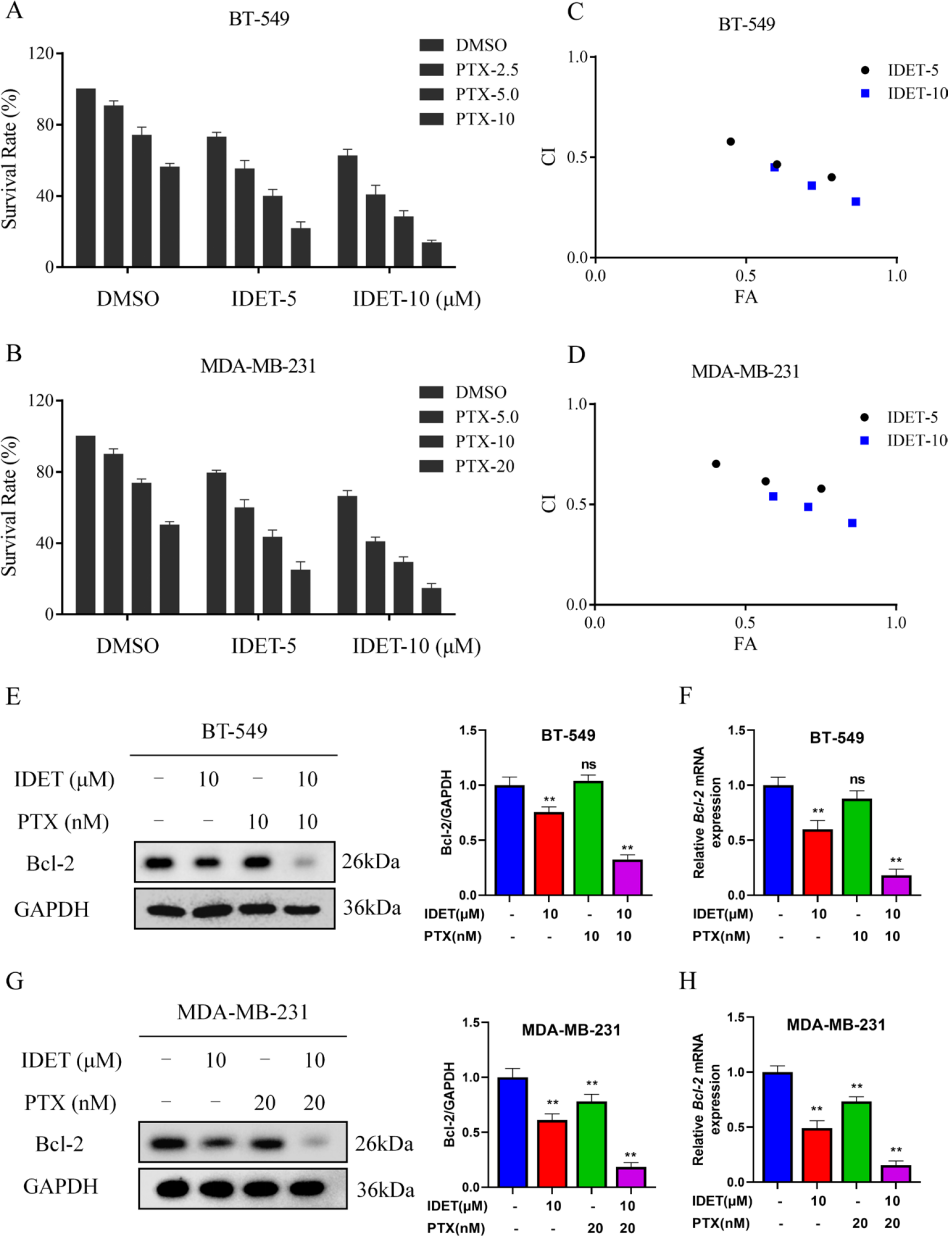
Isodeoxyelephantopin (IDET) enhances the anti‐tumor activity of paclitaxel in triple‐negative breast cancer (TNBC) cells. (A, B) The survival rate of cells treated for 24 h with various concentrations of PTX, either alone or in combination with IDET, was evaluated. (C, D) The corresponding CI values were calculated based on the survival rate results shown in panels A and B. (E–H) The levels of Bcl‐2 in BT‐549 and MDA‐MB‐231 cells were evaluated after treatment with IDET, PTX, or their combination.

To enhance the potential for clinical application, we further evaluated the effectiveness of combining IDET with paclitaxel in inhibiting TNBC cells. Before conducting the combination therapy experiment, we performed a preliminary experiment to assess the activity of paclitaxel on MDA‐MB‐231 and BT‐549 cells. Given the differential sensitivity of these cells to paclitaxel, we selected an appropriate concentration for the subsequent combination therapy experiments. Survival experiments revealed that combining IDET with paclitaxel significantly inhibits cell growth (Figure 4A,B). The combination index values further indicate that IDET and paclitaxel exhibit strong synergistic anti‐tumor activity in TNBC cells (Figure 4C,D). Western blotting and qRT‐PCR results also showed that combining IDET with paclitaxel inhibits Bcl‐2 expression more effectively than using either treatment alone (Figure 4E–H).

### 3.5 The Inhibition of STAT3 Phosphorylation Is Vital for the Synergistic Anti‐Tumor Activity of IDET and Paclitaxel

**FIGURE 5:**
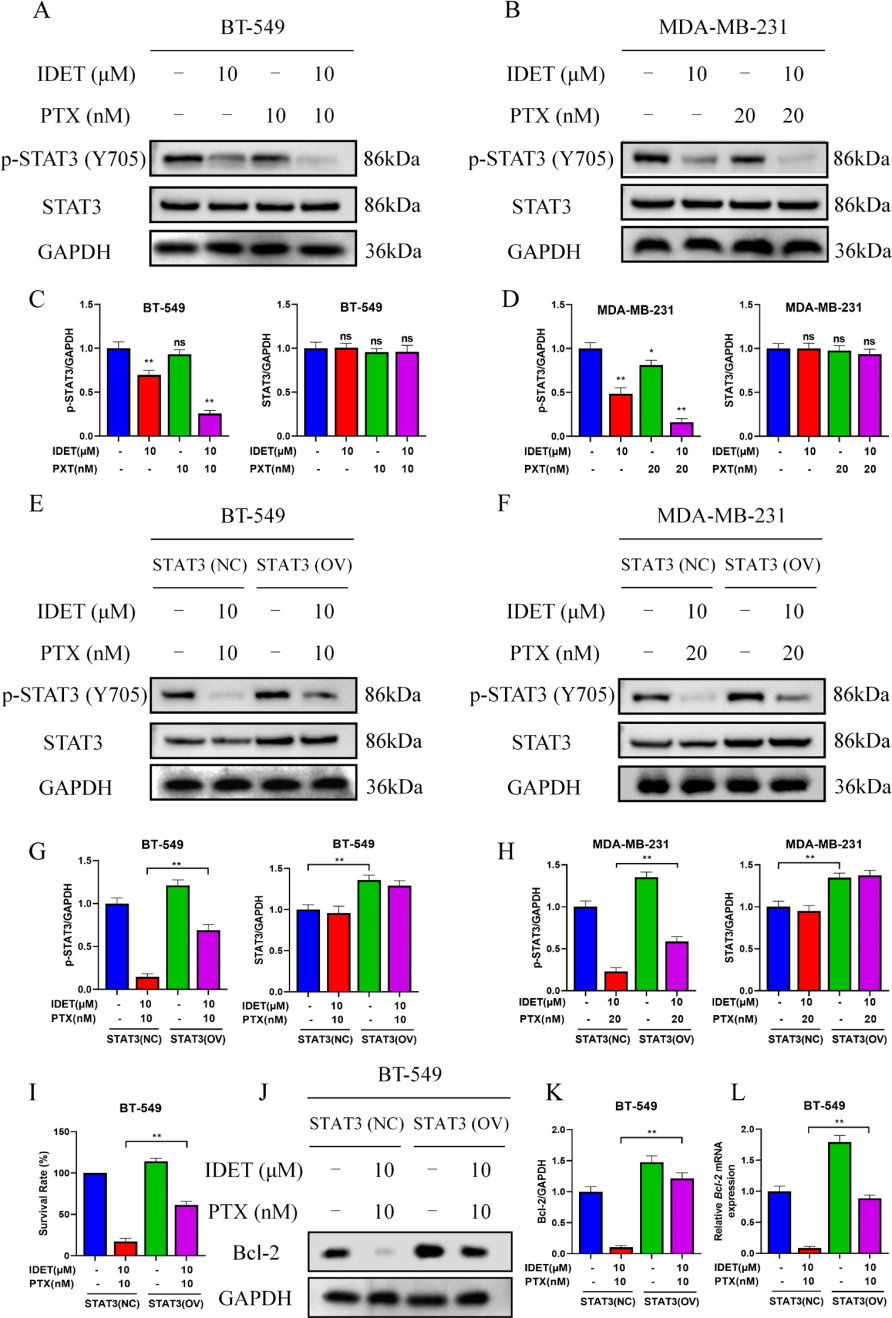
The inhibition of STAT3 phosphorylation is vital for the synergistic anti‐tumor activity of Isodeoxyelephantopin (IDET) and paclitaxel. (A, B) The levels of p‐STAT3 and signal transducer and activator of transcription 3 (STAT3) in BT‐549 and MDA‐MB‐231 cells were assessed following treatment with IDET, PTX, or their combination. (C, D) Statistical analysis of western blot results for A and B. (E, F) The levels of p‐STAT3 and STAT3 in BT‐549 and MDA‐MB‐231 cells (both overexpressing STAT3) were measured following treatment with IDET and PTX. (G, H) Statistical analysis of western blot results for E and F. (I) The survival rate of cells was assessed after treatment with IDET and PTX for 24 h. (J–L) The levels of Bcl‐2 in BT‐549 (overexpressing STAT3) cells were measured following treatment with IDET and PTX.

Considering the better synergistic activity of IDET and paclitaxel, we proceeded to measure the phosphorylation levels of STAT3 in BT‐549 and MDA‐MB‐231 cells following their combined treatment. Western blotting results showed that combining IDET with paclitaxel significantly reduces p‐STAT3 expression in both BT‐549 and MDA‐MB‐231 cells (Figure 5A–D). Importantly, cells with high STAT3 expression showed a marked decrease in sensitivity to the combination treatment, and the reduction in Bcl‐2 levels caused by IDET and paclitaxel was also reversed (Figure 5E–L).

### 3.6 IDET Can Enhance the Anti‐Tumor Activity of Paclitaxel In Vivo

**FIGURE 6:**
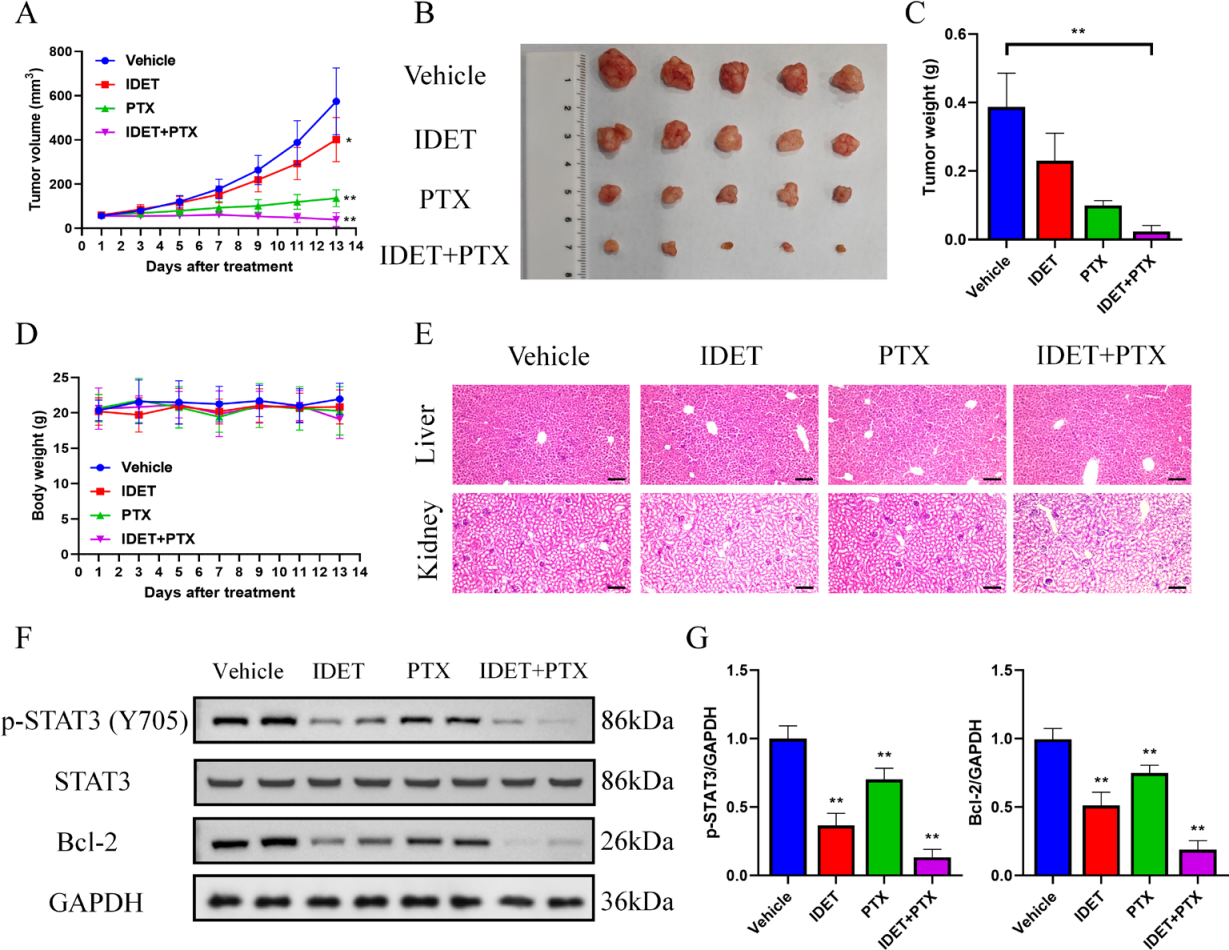
Isodeoxyelephantopin (IDET) enhances the anti‐tumor activity of paclitaxel in vivo. (A, B) The diagram of tumor volume for the Vehicle, IDET, PTX, and the IDET and PTX combination groups. (C, D) Tumor and body weights for the Vehicle, IDET, PTX, and the combination of IDET and PTX groups. (E) HE staining results for the liver and kidney (Scale bar: 100 μm). (F, G) The levels of p‐STAT3, signal transducer and activator of transcription 3 (STAT3), and Bcl‐2 were assessed in the Vehicle, IDET, PTX, and the combination of IDET and PTX groups.

We next evaluated the anti‐tumor activity of IDET combined with paclitaxel in vivo. The results indicate that the combination of IDET and paclitaxel has better anti‐tumor activity compared to monotherapy (Figure 6A–C). Additionally, the weight of the mice and the HE of the liver and kidneys suggest that this combination has good tolerance (Figure 6D,E). Consistent with in vitro studies, the combination of IDET and paclitaxel more effectively inhibits STAT3 phosphorylation and Bcl‐2 expression (Figure 6F,G).

**FIGURE 7:**
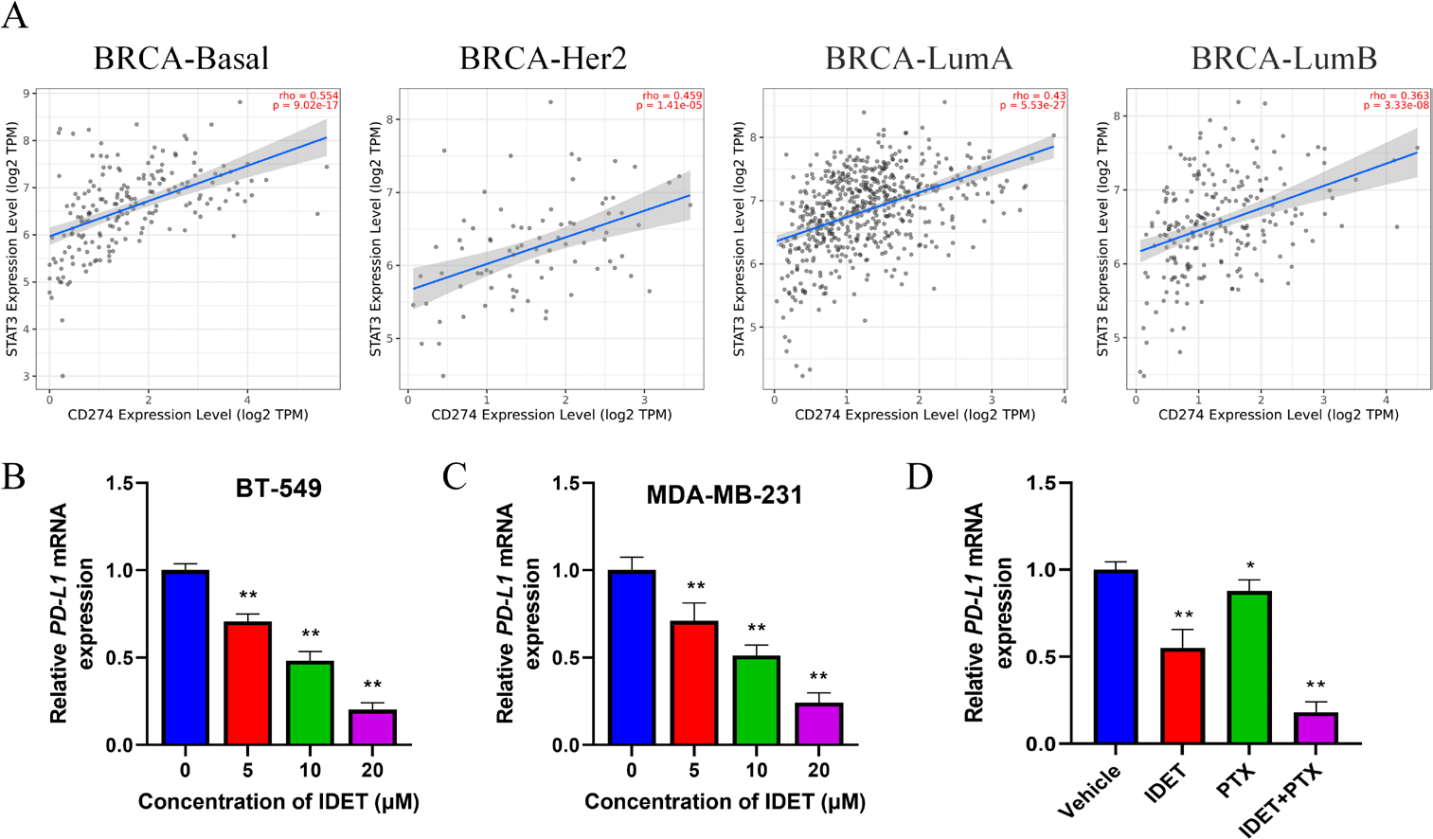
The combination of Isodeoxyelephantopin (IDET) and paclitaxel significantly inhibits PD‐L1 expression in vivo. (A) The correlation between signal transducer and activator of transcription 3 (STAT3) and PD‐L1 (CD274) in breast cancer was assessed using the TIMER2.0 database. (B, C) The mRNA expression levels of PD‐L1 in BT‐549 and MDA‐MB‐231 cells were assessed following treatment with IDET. (D) The mRNA expression levels of PD‐L1 were assessed in the Vehicle, IDET, PTX, and the combination of IDET and PTX groups.

Previous studies have shown that STAT3 is closely related to tumor immunity (Zhang et al. 2022; Dong et al. 2023). Through the TIMER2.0 database, we found that STAT3 was positively correlated with programmed death‐ligand 1 (PD‐L1, CD274) expression in breast cancer, and this correlation was more pronounced in BRCA‐Basal‐type breast cancer (Figure 7A). qRT–PCR results showed that IDET can inhibit the expression of PD‐L1 in a concentration‐dependent manner (Figure 7B,C). Importantly, the combination of IDET and paclitaxel significantly inhibits PD‐L1 expression in vivo (Figure 7D). These results suggest that IDET may have the potential to enhance immune cell‐mediated killing.

## 4 Discussion

TNBC is characterized by the absence of estrogen receptors, progesterone receptors, and human epidermal growth factor receptor 2, rendering it unresponsive to hormone therapies or HER2‐targeted treatments commonly used for other breast cancer subtypes (Bianchini et al. 2022). This makes TNBC treatment more challenging, often requiring a combination of surgery, radiation, and chemotherapy for optimal outcomes (Jiang et al. 2024; Liedtke et al. 2023). Natural products have historically constituted a critical foundation in cancer therapy, offering a diverse range of bioactive compounds recognized for their potential anti‐cancer properties (Mize et al. 2023; Fakhri et al. 2024). The ongoing exploration of natural products continues to be a promising avenue for discovering novel anti‐cancer agents, as they hold the potential to enhance treatment efficacy, reduce side effects, and overcome resistance that can develop with conventional therapies (Ni et al. 2023; Yu et al. 2018; Chao et al. 2019). Our research shows that IDET exhibits significant inhibitory activity against TNBC cells. More importantly, IDET has the potential to enhance the in vivo anti‐TNBC effects of paclitaxel. These findings offer valuable insights and suggest a promising drug combination for the treatment of TNBC.

STAT3 is a key transcription factor that significantly contributes to cancer development and progression by mediating cellular responses to cytokines and growth factors. STAT3 has emerged as a promising target due to its frequent dysregulation in various malignancies, where it often acts as an oncogene, promoting tumor growth, survival, and immune evasion (Dong et al. 2023). Consequently, inhibiting STAT3's activity presents a strategic approach to disrupt these oncogenic pathways and enhance therapeutic efficacy, making it a focal point for developing novel cancer treatments (Arun et al. 2024; Molenda et al. 2023). Upon activation, STAT3 becomes phosphorylated, dimerizes, and translocates to the nucleus, where it drives the transcription of several target genes involved in proliferation and anti‐apoptosis, including Bcl‐2, Cyclin D1, and Mcl‐1 (Khan et al. 2024; Shi et al. 2024). Among these, Bcl‐2 plays a critical role in inhibiting the mitochondrial apoptotic pathway by preventing the release of cytochrome c and subsequent caspase activation. In this study, we first discovered that IDET inhibits STAT3 phosphorylation in TNBC cells, thereby exerting its anti‐tumor activity. Notably, this inhibition led to a significant downregulation of Bcl‐2. Furthermore, overexpression of STAT3 restored Bcl‐2 expression and reversed IDET‐induced cell death, supporting the notion that IDET mediates its anti‐TNBC effects by disrupting the STAT3/Bcl‐2 signaling axis. However, a more detailed investigation into the molecular mechanisms by which IDET regulates STAT3 phosphorylation is still needed for further understanding.

It is noteworthy that IDET can significantly enhance the anti‐tumor efficacy of paclitaxel. Mechanistic studies indicate that the inhibition of STAT3 phosphorylation is vital for the synergistic effects of these two treatments. In vivo experiments confirmed that the co‐administration of IDET and paclitaxel significantly suppresses tumor growth in TNBC models, accompanied by a marked reduction in phosphorylated STAT3 and Bcl‐2 expression, while exhibiting favorable systemic tolerance. These findings establish a theoretical foundation for the combined application of these therapies in the management of TNBC. However, the detailed molecular mechanisms underlying this combination and the specific ways it induces TNBC cell death still require further investigation. Additionally, given that activation of the STAT3 pathway may be closely related to chemotherapy drug resistance, further research into cell resistance, potential resistance mechanisms, and IDET‐based combination therapies is warranted. This represents an important direction for our future work.

Another interesting result is that we found that IDET can significantly inhibit the expression of PD‐L1 in vitro and in vivo. PD‐L1 inhibitors represent a groundbreaking advancement in immunotherapy by targeting the PD‐L1 protein on cancer cells. PD‐L1 binds to PD‐1 receptors on immune cells, effectively turning off their ability to attack tumors. By blocking this interaction, PD‐L1 inhibitors reinvigorate the immune system, enhancing its ability to recognize and destroy cancer cells (Sun et al. 2023; Wang, Dougan, and Dougan 2023). This approach harnesses the body's natural immune responses, offering a potent strategy for treating various cancers that are resistant to traditional therapies (Tang et al. 2023; Marra et al. 2019). The success of PD‐L1 inhibitors marks a significant shift towards more personalized and effective cancer treatments. Our research provides a preliminary basis for combining IDET with chemotherapy and immunotherapy.

## Author Contributions

**Danfeng Lin:** conceptualization (equal), formal analysis (equal), funding acquisition (equal), methodology (equal), software (equal), validation (equal), writing – original draft (equal), writing – review and editing (equal). **Hui Wang:** conceptualization (equal), formal analysis (equal), methodology (equal), validation (equal). **Shixi Wu:** formal analysis (equal), software (equal), validation (equal).

## Conflicts of Interest

The authors declare no conflicts of interest.

## Data Availability

The data presented in the study are available in the article.
